# A Christmas Wish

**Published:** 1918-12

**Authors:** 


### TEXAS MEDICAL JOURNAL

DR. F. E. DANIEL, Founder.

Established July, 1885

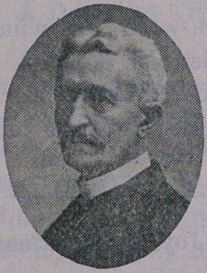

MRS. F. E. DANIEL, Publisher and Managing Editor.

VOL. XXXIV No. 6
AUSTIN, DECEMBER, 1918.

PUBLISHED MONTHLY. Subscription: $1.50

The publisher is not responsible for views of contributors.

ASSISTANT EDITORS:

DR. JOHN S. TURNER, Dallas, Texas. DR. JOE BECTON, Greenville, Texas.

DR. E. B. PARSONS, Palestine, Texas. DR. M. F. BLEDSOE, Port Arthur.

DR. A. P. HOWARD, Houston, Texas. DR. J. H. FLORENCE, Tulsa, Oklahoma.

DR. J. H. REUSS, Cuero, Texas.

ARMY MEDICAL
SEP 23 1947
LIBRARY

### EDITORIAL.

### A Christmas Wish.

May those who have lost loved ones in death during the year coming so soon to a close have the consolation that comes from a realization of the fact that what we call death is but a passing to something better and into a life greater, sweeter by far than anything we have known here, and in which there will be no partings.

May those who have come out of the war maimed and wounded, and the relatives and friends of those, ever feel that the spirit in man can not be crippled except through some act of the man himself, and that, after all, man's spirit is the best part of him.

May we all feel thankful that we have been privileged to have even a small part in making the world a better place in which to live, in alleviating for all time to come much of the distress and suffering that comes from tyranny and oppression, in freeing the world from cruel war and persecutions.

May every American who has contributed anything to winning this war, feel a new pride in his Americanism and a new love for his country that will keep him from ever doing a thing that will discredit in the least the country or himself.

May our hearts in this Christmas-tide go out to the Giver of all Good and to all his creatures in love and tenderness, anr may we feel as we have never felt, the Fatherhood of God and the Brotherhood of man.

May our daily lives reflect that feeling.

"Heap on more wood! the wind is chill;
But let it whistle as it will—
We'll keep our Christmas merry still"—
If you will pay that little bill.

